# Durum Wheat Cultivars Express Different Level of Resistance to Granary Weevil, *Sitophilus granarius* (Coleoptera; Curculionidae) Infestation

**DOI:** 10.3390/insects11060343

**Published:** 2020-06-03

**Authors:** Darija Lemic, Katarina M. Mikac, Matej Genda, Željko Jukić, Ivana Pajač Živković

**Affiliations:** 1 Department for Agricultural Zoology, Faculty of Agriculture, University of Zagreb, 10000 Zagreb, Croatia; matej.genda44@gmail.com (M.G.); ipajac@agr.hr (I.P.Ž.); 2 Centre for Sustainable Ecosystem Solutions, School of Earth, Atmospheric and Life Sciences, Faculty of Science, Medicine and Health, University of Wollongong, 2522 Wollongong, Australia; kmikac@uow.edu.au; 3 Department of Field Crops, Forage and Grassland, Faculty of Agriculture, University of Zagreb, 10000 Zagreb, Croatia; zjukic@agr.hr

**Keywords:** *Sitophilus granarius* L., storage pest, durum wheat, progeny, resistance

## Abstract

The granary weevil, *Sitophilus granarius* Linnaeus 1875, is a primary pest of stored grains worldwide. Feeding damage and progeny production of *S. granarius* was estimated to identify the levels of resistance of the insect on different durum wheat cultivars. Insect attack on four different durum wheat cultivars was investigated over a period of 20 weeks. Durum wheats were artificially infected with 20 individuals of *S. granarius*. Every two weeks the sample weight, hectoliter weight, moisture and the number of live weevils, including their number of progenies, were recorded. Overall findings revealed different levels of resistance of different durum wheat cultivars to *S. granarius* infestation. The Primadur cultivar had the highest resistance, followed by the Marco Aurelio and Cesare cultivars followed finally by the Tito Flavio cultivar which was highly susceptible to *S. granarius*. For all cultivars, apart from Primadur, *S. granarius* metabolism increased humidity and temperature, leading to grain degradation and resulting in the potential complete loss of market value if under field conditions. Evidently, durum wheat characteristics affect the life cycle of *S. granarius*, primarily their progeny, and thus the damage they undertake to the wheat itself. These findings are important because they enable the strategic selection of wheat cultivars that can be stored for a longer time period, while more sensitive wheat cultivars can be selected for shorter storage time and thus faster delivery to market.

## 1. Introduction

Durum wheat (*Triticum durum* Desf.) cultivars are very important in the global production of wheat. Due to their specific properties, such as high protein content and gluten, this variety of grain is of a reliable quality and thus favored by the global food industry. This variety is grown across approximately 17 million hectares resulting in 37 million tonnes of wheat annually [1]. Production can vary from 32 to 42 million tonnes because of local prevailing environmental factors. Globally, 8% of wheat production is of the durum wheat cultivar [1,2]. The main durum-producing countries and trading blocs include the European Union, North America, Turkey, Algeria and Kazakhstan. Minor wheat for export production countries include Syria, Morocco, Tunisia, India, Australia, Argentina and Chile [2]. In the EU, major wheat producers include Italy, France, Greece, and Spain [2]. Although durum wheat is a relatively minor crop worldwide, it is the main crop within the Mediterranean basin and makes up the raw material for numerous manufactured and internationally traded products, such as pasta and couscous [3,4,5,6,7,8].

Stored grains can affect insect pest attacks because of the lack of vital nutrients or the presence of chemical compounds that adversely affect insect development [8,9]. The physico-chemical properties of wheat grain such as grain color, hardness index, seed size, gluten index, Zeleny sedimentation volume, and gluten viscoelasticity are a function of mainly genetic and partly environmental factors [10,11]. Knowledge of all of the above-mentioned parameters of wheat can help to understand the influence that these have on the development rate of feeding insects. Knowledge of the physico-chemical characteristics and their relationship with *Sitophilus granarius* infestation can be used to inform integrated management of stored wheat [12,13].

*Sitophilus granarius* L. (Coleoptera: Curculionidae) is the most common insect species found in smaller rural warehouses and in large storage silos [14,15,16]. The *S. granarius* is very destructive since both the adult and larvae of the species attack stored grains (e.g., wheat, barley, rye, oats, corn, rice) [17]. In addition to grain, *S. granarius* also attacks milled flour, sows, groats and manufactured pasta. Infestation by this species causes severe quantitative and qualitative losses due to direct insect feeding on grains, alteration of nutritional and aesthetic value and contamination of commodities with insect bodies, excrement and mycotoxins that result from insect-promoted fungal growth during storage [18,19,20,21]. Adults cause damage by destroying kernels, mainly the germ, producing grain debris, raising grain temperature and water content, and facilitating the invasion of secondary insect pests (i.e., psocids, mites, bacteria, and fungi) [22]. It is well known that *S. granarius* larvae develop inside the kernel and consume about 64% of its content [23,24]. A single pair of beetles, under optimal conditions, could result in hundreds of thousands of offspring in a year. It is therefore understandable that the damage caused by this insect can be very high [14]. Under natural and fluctuating storage conditions, the pest can have three to five generations/year and it is the abiotic parameters (temperature and relative humidity) that have the greatest influence on *S. granarius* development rate and population increase [17]. The insect resistance mechanisms of cereal grains are complex and depend on biochemical and physical adaptation of storage insects to these properties [11,25,26,27,28]. The expression of insect resistance in different plant tissue varies tremendously during the lifetime of a plant [29]. Host-plant resistance plays an important role in the management of insect pests of wheat. Insect development of wheat resistance represents some of the earliest examples of host-plant resistance. A range of breeding methods, from traditional evaluation and selection to marker-assisted selection, has been used to develop host-plant resistance in wheat [29]. While there are many findings of host-plant resistance on cereals (including soft wheat) to different insect pests (Hessian fly, *Mayetiola destructor* (Say) [30,31]; wheat stem sawfly, *Cephus cinctus* Nort. [32]; wheat midge, *Sitodiplosis mosellana* (Géhin) [33,34,35]; Russian wheat aphid, *Diuraphis noxia* (Mordvilko) [36,37,38]; English grain aphid, *Sitobion avenae* (Fabricius) [39,40,41,42]; bird cherry-oat aphid, *Rhopalosiphum padi* (L) [41,43]; greenbug, *Schizaphis graminum* (Rondani) [44,45]), there is no published data available on grain resistance to insects during storage.

Each durum wheat variety’s commercial seed producer clearly states knowledge of resistance to physical properties of plant and especially to fungal diseases, however, there is no publicly available data on the resistance or susceptibility of a particular cultivar to *S. granarius* or other important insect storage pests. The aim of this study was therefore to determine the level of resistance of different durum wheat cultivars to *S. granarius* infestation over time.

## 2. Materials and Methods

### 2.1. Experimental Design

The experiment was undertaken from November 2018 to March 2019 at the Faculty of Agriculture, University of Zagreb, Croatia. Here we used a completely randomized experimental design [46,47,48,49]. Four durum wheat cultivars were used in the study: Tito Flavio, Marco Aurelio, Cesare and Primadur. Commercial characteristics of each cultivar are presented in Table 1 [27,28,50]. In summary, all durum wheat cultivars available for this study have excellent productivity, high protein content, good-to-high yellow index in semolina, wide adaptability, and resistance to *Septoria*.

Grains used in experiments were cleaned of straw, chaff, light grains and other impurities before use in experiments. Kernels with any form of damage were removed and samples were sterilized to kill any live insects originating from natural infestations. Samples were then acclimatized in woven cotton bags at room temperature (20 ± 2 °C and 25%–30% relative humidity). The initial moisture content of seeds was determined using the oven method [51]. To achieve the desired 13% moisture level, the rewetting formula was used. The four durum wheat cultivars, with four replicates for each cultivar (total: 16 replicates), were tested across the experimental time period of 20 weeks. Each of the four replicates had 250 g of grain added at the start of the experiment. Initial readings of moisture (%), weight (g) and hectoliter mass (kg/l) in a volume of 210 mL were obtained using a grain moisture meter (Draminski S.A., Olsztyn, Poland).

After these initial measurements, each replicate (of 250 g) was infested with 20 each 10-day-old adult *S. granarius* (10 males and 10 females separated by sex according to the methods of Dinuta et al. [52]). In total, 16 durum wheat replicates were infected with a total of 320 adult *S. granarius*. This experimental design is in accordance with numerous other published studies investigating aspects of durum wheat host preferences [47,53,54] and cultivar resistance to insect attack [49]. These studies were undertaken as controlled experiments (i.e., non-seasonal), use a completely randomized design, and have up to four cultivars and four replicates for their experiments.

### 2.2. Data Collection and Analysis

Across a period of 20 weeks, nine evaluations were performed every 14 days. During each evaluation, the physical properties of the grain including grain moisture (%), weight (g), and hectoliter mass (kg/hl) were measured using a Draminski device. The number of live and dead *S. granarius* was also recorded. After the final evaluation, relative humidity (%) and air temperature (°C) inside of the experimental containers using thermometer and hygrometer were also measured. Visual, olfactory and tactile observations of sample quality were recorded. The indicators used by a single observer were: unpleasant odors emanating from decaying grain, condensation on the ceiling of the storage container, excessive moisture on the base or on the sides of the containers, visible molds and diseased grain [55,56,57,58].

Grain weight, moisture, hectoliter mass and number of live *S. granarius* in each replication of each cultivar were recorded over the 20-week investigation period. The data collected was analyzed using a repeated measures ANOVA to determine the difference in the resistance of durum wheat cultivars to *S. granarius* infestation and the differences occurred in each cultivar among the 14-day evaluation periods. A post-hoc means test was used when significant differences were found (Tukey’s HSD). These analyses were performed using ARM 2019^®^ GDM software [59].

## 3. Results

During the first eight weeks of the experiment, the number of *S. granarius* varied between cultivars but not significantly. During the next experimental time period (10–18 weeks) the Tito Flavio cultivar was found to be susceptible to *S. granarius* population increases. In the 14th week of the experiment the number of insects increased seven times more on the Tito Flavio cultivar. At the 20-week time period the Tito Flavio cultivar remained the most susceptible cultivar to the development and progeny production of *S. granarius*, followed by the Cesare and Marco Aurelio cultivars. The Primadur cultivar showed resistance to *S. granarius* across the whole experimental time period. The number of *S. granarius* remained lower than that of the initial infestation for the duration of experimental period and total mortality was observed during the 16th week of the experiment (Table 2). In the cultivar Tito Flavio during the experiment 20-week, the number of *S. granarius* varied significantly. The maximum number of *S. granarius* progeny were noted in the 14th week.

Of the four durum wheat cultivars investigated, *S. granarius* caused a significant weight loss to cultivar Tito Flavio (25%), followed by cultivar Marko Aurelio (16%). In the cultivars Cesare (7%) and Primadur (2%), weight loss was observed but it was not significantly different from the initial weight. All cultivars differed significantly in their final weights (Table 3).

Grain moisture significantly increased after the 14th week in the cultivar Tito Flavio. During the final weeks of the experiment, grain moisture increased by 45% in Tito Flavio and 2% in Marko Aurelio. The cultivars Cesare and Primadur showed slight changes in grain moisture across the experimental period. Only Tito Flavio differed significantly in its final moisture content compared with the other cultivars (Table 4).

Hectoliter mass significant differences were found after *S. granarius* fed on grain in each cultivar. The highest significant hectoliter mass was found for the Primadur cultivar, which also had the lowest loss of only 5% overall. The lowest significant hectoliter mass was observed for the Tito Flavio cultivar with a decrease of 23%. The cultivars Marco Aurelio and Cesare decreased by 14.5% and 15.5% hectoliter mass respectively, though they were not significant (Table 5).

Finally, ambient temperature and relative humidity inside of all of the experimental containers measured at the end of experiment showed high microbiological activity and insect metabolism. Temperature was the lowest and most significant in the Primadur cultivar; other cultivars were not significantly different for this variable. Humidity was highest and most significant in the Tito Flavio (73%), followed by the Cesare (66%) and Marco Aurelio (54%) cultivars, and the lowest humidity was observed in the Primadur cultivar (28%) (Table 6).

Primadur showed the greatest resistance to *S. granarius* infestation as there were no observed changes in grain quality indicators during the whole 20-week experimental period. The cultivars Marco Aurelio and Cesare displayed slight-to-medium changes in grain quality because of *S. granarius* progeny production and metabolism. These two cultivars were deemed moderately resistant to *S. granarius*. Finally, Tito Flavio showed the highest susceptibility to *S. granarius* which resulted in the total destruction of grains at the end of the 20-week experimental period (Table 7). Essentially, every wheat kernel of Tito Flavio durum wheat cultivar in all four jars was severely damaged by *S. granarius* infestation in our experiment.

## 4. Discussion

*Sitophilus granarius* is universally regarded as one of the most destructive primary pests of stored wheat [33,34] and we aimed to determine the level of resistance of different durum wheat cultivars to this insect. During this study we investigated the progeny potential of this pest on durum wheat cultivars present on market and in the process produced data to demonstrate that the four durum wheat cultivars tested exhibited varying levels of resistance to *S. granarius.*

Cereal grains’ resistance mechanisms to insect attack are complex and depend on physico-chemical and biochemical properties of the grains themselves and on the subsequent biochemical and physical adaptation of postharvest insects to them. Stored wheat (grain) may have high resistance to insects because of the lack of vital nutrients provided and/or the presence of compounds that negatively affect insect development [8]. Grain, in contrast to plant tissue, does not possess insect-defensive compounds such as alkaloids, saponins, non-protein amino acids, terpenoids or phenols [60,61,62]. Its chemical composition and nutritional quality for insects does not radically change during storage. Grain feeding insects, therefore, have a stable food supply without specific defensive compounds changing during storage and insect development [60].

Few studies have compared the nutrition value of soft and durum wheat cultivars for *Sitophlus* spp. [8,60,63,64]. Though some have found higher infestation and greater abundance of *S. granarius* on soft wheat compared with durum wheat cultivars [65,66], this could be because the main digestive enzyme for *S. granarius* is amylase [67]. Nawrot et al. [60] found that seven times more amylase was present in *S. granarius* fed on soft wheat cultivars than on durum wheat cultivars. It is believed that the enzyme inhibitors impede digestion through their action on insect gut digestive-amylases and proteases, which play a key role in the digestion of plant starch and proteins [5,8,63,68,69]. Enzyme inhibitors from plants could be promising candidates for new biocontrol agents [70].

In this experiment, the durum wheat cultivar Primadur was found to be unfavorable for *S. granarius* survival. One of the few studies conducted on the Primadur cultivar showed that this cultivar was highly disease resistant; the authors, however, did not investigate or declare the cultivar’s insect resistance [27]. On this cultivar, *S. granarius* did not cause any significant damage during the whole 20-week investigation period and did not produce second generation offspring. The number of live *S. granarius* from the initial infection gradually decreased, and after 16 weeks there were no live *S. granarius* in any replicate. Interestingly, in one replicate of the Primadur cultivar, 38 live *S. granarius* were observed, suggesting successful reproduction, but eight weeks later all these individuals were dead. More intensive laboratory studies of the physico-chemical properties of the Primadur cultivar are needed to precisely determine cause and effect of the cultivar’s physico-chemical characteristics on insect mortality.

For the Tito Flavio cultivar, 100% grain destruction caused by *S. granarius* metabolism was observed. *Sitophilus granarius* numbers across the 20 weeks climbed from an initial number of 20 to 300 of individuals in the most infected replicate. Sample weight reduced by 25% during this period, and hectoliter weight dropped to 60 kg/hl; below the minimum quality value needed to meet market requirements. During this point in the experiment, the grain became completely unusable with the lowest visual, olfactory and tactile quality parameters recorded. In the Marco Aurelio cultivar, *S. granarius* in two of the four replicates died, while in the remaining two replicates the number of *S. granarius* increased during the experimental time period. As expected, where the number of *S. granarius* increased, so did grain moisture. This occurred likely because of *S. granarius*’ metabolic processes [71]. Informative data were also obtained when measuring the humidity and ambient temperature of experimental containers with wheat grain at the end of the study. With their diet and metabolism, *S. granarius* warmed the closed container system to above 27 °C and raised the moisture levels >70%. It is likely that these measurements would have been higher except that all containers had vents. Under these conditions, the expected development of mold and the unpleasant odors of completely destroyed grain were observed.

## 5. Conclusions

There was a difference in durum wheat cultivar resistance, hence certain grain characteristics can affect the life cycle of *S. granarius*, its progeny production and the damage it causes. This was especially evident for the Primadur cultivar which was found to be highly resistant to *S. granaries*. Future studies should investigate the physical and biochemical properties of particular durum wheat cultivars to determine the parameters that are important in the adaptation of *S. granarius* to nutrition and the resulting breeding potential. Identifying “resistance” factors that can stop the development and progeny of the first generation of insect pests can be key information to improve integrated pest management (IPM) in storage structures.

## Figures and Tables

**Table 1:** Commercial characteristics of durum wheat cultivars.

Morpho-physiological Characteristics	Durum Wheat Cultivars			
	Tito Flavio	Marco Aurelio	Cesar	Primadur
**Earing time**	Medium-Late	Medium	Medium-late	Medium
**Plant size**	Medium	Medium	Medium	Medium
**Resistance**				
**Lodging**	Excellent	Good	Excellent	Excellent
**Cold**	Excellent	Good	Good	Good
**Powdery mildew**	Good	Good	Good	Excellent
**Brown rust**	Excellent	Good	Good	Good
***Septoria***	Excellent	Excellent	Medium	Excellent
***Fusarium*** **spp.**	Good	Good	Excellent	Medium
**Quality characteristics**				
**Thousand kernel weight**	50–57 g	53–58 g	45–50 g	46–50 g
**Hectoliter weight**	High	High	Excellent	High
**Yellow index**	Good	High	Excellent	High
**Protein content**	Good	Excellent	High	High
**Gluten tenacity**	Medium	High	Good	High

**Table 2:** The average number (± SE) of living adult *S. granarius* in durum wheat cultivars during the 20-week experimental time period.

Evaluation Period	Durum Wheat Cultivars				HSD ^1^ *p* = 0.05 ***
	Marco Aurelio	Cesare	Primadur	Tito Flavio	
**1**	20 *	20	20	20	-
**2**	1.5 ± 1.5	9.3 ± 3.5	9.5 ± 3.6	5.3 ± 3.9 BC	13.33
**3**	3.5 ± 3.5	6.3 ± 2.8	9.5 ± 3.4	4.0 ± 2.0 C	12.60
**4**	21.3 ± 20.6	55.5 ± 35.8	11.3 ± 9.1	102.3 ± 31.2 ABC ^2^	114.40
**5**	35.5 ± 33.2	67.5 ± 45.0	11.3 ± 9.7	108.3 ± 26.8 ABC	137.41
**6**	4.9 ± 0.5 ab ^1^^,^**	32.3 ± 0.3 ab	1.9 ± 0.3 b	92.4 ± 0.01 a, ABC	90.33
**7**	61.8 ± 47.4	68.8 ± 44.0	4.3 ± 2.4	147.8 ± 62.5 A	169.47
**8**	75.0 ± 59.5 ab	97.0 ± 47.8 ab	0.0 ± 0.0 b	133.8 ± 44.5 a, AB	131.65
**9**	60.3 ± 47.6 ab	107.0 ± 34.7 ab	0.0 ± 0.0 b	121.3 ± 51.4 a, ABC	194.2
**HSD**^2^***p*=0.05** ***	35.75	50.72	21.34	118.31	

* Initial infestation (20 *S. granarius* per replicates); ** Mean values of the same row followed by the same letter (a, b, ab) were not significantly different (*p* ≥ 0.05; HSD test); *** HSD was determined by comparing the average *S. granarius* number in durum wheat cultivars for each two-week evaluation period; ^1^ small letters refer to differences among cultivars: the data was log (*x* + 1) transformed; ^2^ capital letters refer to differences among evaluation periods: the data were arcsin transformed √x.

**Table 3:** Durum wheat (± SE) grain weight (g) across the 20-week experimental time period.

EvaluationPeriod	Durum Wheat Cultivars				HSD ^1^ *p* = 0.05 **
	Marco Aurelio	Cesare	Primadur	Tito Flavio	
**1** **^¥^**	170.43 ± 2.3 C ^2^	177.23 ± 2.3	179.6 ± 3.4	175.08 ± 2.6 B	11.59
**2**	167.18 ± 0.6 b ^1^^,^*, A	177.95 ± 1.4 ab	179.4. ± 1.2 a	169.88 ± 3.3 b, A	4.19
**3**	166.78 ± 2.1 b, AB	176.6 ± 0.8 a	175.4 ± 2.9 a	169.65 ± 1.4 ab, A	8.56
**4**	156.88 ± 1.9 ABC	175.58 ± 2.4	173.0 ± 2.1	168.8 ± 2.9 AB	9.83
**5**	156.93 ± 2.1 ABC	178.68 ± 4.7	175.15 ± 7.6	158.2 ± 8.2 A	27.09
**6**	156.08 ± 0.0 ABC	174.78 ± 0.0	177.62 ± 0.0	154.96 ± 0.0 A	28.04
**7**	155.78 ± 4.8 ABC	171.18 ± 8.3	177.6 ± 6.2	154.73 ± 9.6 A	30.98
**8**	145.58 ± 7.6 b, BC	171.9 ± 1.2 ab	177.6 ± 6.2 a	134.38 ± 12.9 b, A	40.61
**9**	143.34 ± 9.2 b, ABC	165.65 ± 5.2 ab	177.5 ± 6.0 a	131.65 ± 14.8 b, A	55.53
**HSD**^2^***p*=0.05** **	21.23	22.03	20.48	37.17	

* Mean values of the same row followed by the same letter (a, b, ab) were not significantly different (*p* ≥ 0.05; HSD test); ** HSD was determined by comparing the average weight (four replicates) between wheat durum cultivars for each two-week evaluation period; ^1^ small letters refer to differences among cultivars: data were log (*x* + 1) transformed; ^2^ capital letters refer to differences among evaluation periods: data were arcsin transformed √x. **^¥^** initial bioassay.

**Table 4:** Durum wheat (± SE) grain moisture across the 20-week experimental period.

Evaluation Period	Durum Wheat Cultivars				HSD ^1^ *p* = 0.05 **
	Marco Aurelio	Cesare	Primadur	Tito Flavio	
**1** ^¥^	13.5 ± 0.0 c ^1,^*	13.78 ± 0.0 ab, A ^2^	13.6 ± 0.1 bc, A	13.9 ± 0.0 a, B	0.22
**2**	11.85 ± 0.1 c	12.6 ± 0.0 a, AB	12.33 ± 0.1 b, AB	12.78 ± 0.2 a, B	0.08
**3**	11.68 ± 0.2	12.38 ± 0.2 AB	11.88 ± 0.1 B	12.53 ± 0.3 B	0.99
**4**	11.68 ± 0.3	12.3 ± 0.1 AB	11.85 ± 0.2 B	12.58 ± 0.4 B	1.35
**5**	11.65 ± 0.2	11.6 ± 0.4 AB	11.53 ± 0.4 B	13.18 ± 0.7 B	2.24
**6**	11.72 ± 0.0	11.82 ± 0.0 AB	11.16 ± 0.0 B	12.8 ± 0.0 B	3.53
**7**	12.00 ± 0.6	12.15 ± 1.3 AB	10.95 ± 0.5 B	15.08 ± 2.4 B	4.06
**8**	13.45 ± 1.3 b	11.10 ± 0.0 b, B	10.95 ± 0.5 b, B	24.50 ± 0.9 a, A	3.05
**9**	13.8 ± 2.8 b	11.6 ± 0.6 b, AB	10.95 ± 0.5 b, B	25.25 ± 0.6 a, A	6.18
**HSD**^2^***p*=0.05** **	4.45	2.31	1.42	4.34	

* Mean values of the same row followed by the same letter (a b, ab, c) were not significantly different (*p* ≥ 0.05; HSD test); ** HSD was calculated by comparing the average grain moisture (four replicates) between wheat durum cultivars for each two-week evaluation period; ^1^ small letters refer to differences among cultivars: data were log (*x* + 1) transformed; ^2^ capital letters refer to differences among evaluation periods: data were arcsin transformed √x. **^¥^** initial readings.

**Table 5:** Durum wheat (± SE) grain hectoliter mass across a 20-week experimental period.

Evaluation Period	Durum Wheat Cultivars				HSD ^1^ *p* = 0.05 **
	Marco Aurelio	Cesare	Primadur	Tito Flavio	
**1** **^¥^**	81.43 ± 0.2 ab ^1,^*^,^ A^2^	83.17 ± 0.5 a, A	85.02 ± 0.6 a, A	78.68 ± 1.1 b, A	4.06
**2**	81.25 ± 0.3 b, A	83.1 ± 0.7 ab, A	84.78 ± 0.6 a, A	78.18 ± 1.6 b, A	2.05
**3**	79.83 ± 0.3 ab, AB	82.6 ± 1.1 ab, A	84.28 ± 1.1 a, AB	78.23 ± 1.6 b, A	5.32
**4**	76.75 ± 1.3 ABC	77.6 ± 1.5 AB	79.23 ± 1.0 C	76.75 ± 1.2 A	6.12
**5**	78.23 ± 1.0 ABC	77.95 ± 3.2 AB	80.7 ± 1.7 BC	76.9 ± 4.0 A	15.97
**6**	78.3 ± 0.0 ABC	77.55 ± 0.0 AB	79.98 ± 0.0 BC	75.37 ± 0.0 A	13.12
**7**	73.7 ± 1.7 ABC	76.43 ± 4.6 AB	80.6 ± 1.3 C	70.65 ± 4.2 AB	11.20
**8**	71.28 ± 3.3 ab, BC	74.85 ± 3.4 a, AB	80.6 ± 1.3 a, C	60.73 ± 1.6 b, B	9.62
**9**	69.58 ± 4.0 ab, C	70.28 ± 4.0 ab, B	80.6 ± 1.3 a, C	60.7 ± 1.6 b, B	11.34
**HSD**^2^***p*=0.05** **	9.18	11.41	3.64	11.93	

* Mean values of the same row followed by the same letter (a, b, ab) were not significantly different (*p* ≥ 0.05; HSD test); ** HSD was determined by comparing the average hectoliter mass (four replicates) between wheat durum cultivars for each two-week evaluation period); ^1^ small letters refer to differences among cultivars: data were log (*x* + 1) transformed; ^2^ capital letters refer to differences among evaluation periods: data were arcsin transformed √x. **^¥^** initial reading prior to experimentation.

**Table 6:** The average (± SE) humidity and temperature according to durum wheat cultivars.

Climate Condition	Durum Wheat Cultivars				HSD *p* = 0.05 **
	Marco Aurelio	Cesare	Primadur	Tito Flavio	
**Temperature**	27 ± 0.4 a *	26.2 ± 0.2 a	22.5 ± 0.4 b	26.2 ± 0.5 a	2.38
**Humidity**	53.5 ± 2.5 b	66 ± 2.7 ab	28 ± 0.7 c	72.6 ± 2.7 a	17.11

* Mean values of the same row followed by the same letter (a, b, ab, c) were not significantly different (*p* ≥ 0.05; HSD test); ** HSD was determined by comparing the average humidity and temperatures in closed containers of wheat durum cultivars: data were arcsin transformed √x.

**Table 7:** Visual, olfactory and tactile observation of durum wheat cultivars after 20 weeks of experimentation.

Indicators	Durum Wheat Cultivars			
	Marco Aurelio	Cesare	Primadur	Tito Flavio
**Unpleasant odors**	++	++	-	+++
**Condensation on top**	++	+++	-	+++
**Excessive moisture**	++	+++	-	+++
**Visible molds**	+	++	-	+++
**Diseased tissues**	+	++	-	++

- no observed changes; + slight differences observed on grain (20%) and containers; ++ medium changes observed on grain (50%) and container; +++ high changes observed on grain (100%) and container.
